# Risk assessment of cardiac arrhythmias in the early post-COVID-19 period in non-hospitalized patients—long-term data from the PoLoCOV-CVD study

**DOI:** 10.1038/s41598-026-47954-0

**Published:** 2026-04-16

**Authors:** Joanna Kapusta, Mateusz Babicki, Żaneta Kałuzińska-Kołat, Damian Kołat, Piotr Jankowski, Dariusz A. Kosior, Michał Chudzik

**Affiliations:** 1 Department of Internal Diseases, Rehabilitation and Physical Medicine, Medical University of Lodz, Kosciuszki 4, 90-419 Lodz, Poland; 2 Department of Family Medicine, Wroclaw Medical University, Wroclaw, Poland; 3 Department of Biomedicine and Experimental Surgery, Medical University of Lodz, Lodz, Poland; 4 Department of Functional Genomics, Medical University of Lodz, Lodz, Poland; 5 Department of Internal Medicine and Geriatric Cardiology, Medical Centre for Postgraduate Education, Warsaw, Poland; 6 Mossakowski Medical Research Centre, Polish Academy of Science, Warsaw, Poland; 7 Chair of Sports Medicine, Medical Centre for Postgraduate Education, Warsaw, Poland; 8 Department of Nephrology, Hypertension and Family Medicine, Medical University of Lodz, Lodz, Poland

**Keywords:** SARS-CoV-2, COVID-19, Cardiac arrhythmias, Myocardial damage, Cardiovascular system diseases, Cardiology, Diseases, Medical research

## Abstract

Many studies have shown that SARS-CoV-2 infection requiring hospitalization may result in myocardial damage, heart failure or cardiac arrhythmias, which leads to a severe course of the disease, worsens the prognosis, and is an independent risk factor for death. The study aimed to assess whether the COVID-19 severity affects the occurrence and type of cardiac arrhythmias among non-hospitalized patients without previous CVD in the early period after SARS-CoV-2 infection. The study included patients with no history of prior CVD who suffered from COVID-19 and were treated on an outpatient basis. Study subjects were divided into two groups based on the severity of their COVID-19 course. In the post-COVID period (average 12 ± 6 weeks), patients underwent a standard 12-lead resting electrocardiogram (ECG) and a 24-h Holter monitoring. The analysis included 893 patients, among whom 25.2% had a severe course of COVID-19. Age (*p* = 0.02) and cardiac dysfunction [ejection fraction < 50% and/or contractile dysfunction on echocardiography (*p* = 0.01)] were associated with a higher risk of cardiac arrhythmias after COVID-19. The most commonly observed ECG abnormalities were prolonged QRS complex duration (*p* = 0.035) and the occurrence of supraventricular extrasystoles (*p* = 0.037). Multivariate regression analysis showed that independent risk factors associated with the occurrence of cardiac arrhythmias in non-hospitalized patients after COVID-19 include age, systolic and diastolic LV diameter, LA diameter, and the presence of at least one chronic disease. Based on the analysis of the obtained results, it was observed that the occurrence of cardiac arrhythmias in the early post-COVID-19 period among non-hospitalized patients without prior CVD does not depend on COVID-19 severity. However, identifying significant ventricular arrhythmias should prompt echocardiographic evaluation due to the high risk of left ventricular dysfunction.

**Supplementary Information:**

The online version contains supplementary material available at 10.1038/s41598-026-47954-0.

## Introduction

The emergence of Coronavirus Disease 2019 (COVID-19) caused by the highly infectious Severe Acute Respiratory Syndrome Coronavirus 2 (SARS-CoV-2) became a major worldwide threat to public health four years ago^1^. In most cases, the infection presents with mild cold symptoms such as fever, runny nose, cough or fatigue^2^. Despite its seemingly mild course, COVID-19 should not be underestimated because patients who have recovered, regardless of whether the infection was mild or severe, may develop long-term complications known as “long-COVID” or “post COVID-19 syndrome”^3–5^. According to the World Health Organization, post-COVID-19 condition affects individuals with a history of presumed or confirmed SARS-CoV-2 infection, usually 3 months after the onset of COVID-19, with symptoms that last at least 2 months and cannot be explained by any alternative diagnosis. Typically reported symptoms in post-COVID-19 patients are fatigue, periodic headaches, memory and concentration problems, or anxiety. Still, cardiopulmonary symptoms such as chest pain, shortness of breath, or cardiac arrhythmias are equally common^6^.

Since the beginning of the pandemic, the most common extrapulmonary manifestations of SARS-CoV-2 infection have been cardiovascular disorders, such as myocardial infarction or myocarditis, which lead to cardiomyopathy, bradyarrhythmia or tachyarrhythmia, as well as heart failure^7–9^. Literature data report that in approximately 10–50% of patients, symptoms such as palpitations, shortness of breath, deterioration of physical performance, and heart rhythm disturbances may persist for several months after acute SARS-CoV-2 infection^10–12^. Cardiac arrhythmias associated with COVID-19 were initially described by Wang et al., who reported 17% of arrhythmia cases among patients treated in the intensive care unit^13^. In the Coromilas et al. study conducted on a multinational group of 4526 hospitalized patients, cardiac arrhythmias due to COVID-19 were observed in 18.27% of the subjects^14^. This problem applies not only to individuals in the acute phase of the disease but also after COVID-19, as confirmed by research conducted in a group of 93 patients after COVID-19, where cardiac arrhythmia was observed in 30.1% of subjects^15,16^. Unfortunately, the mechanisms underlying cardiac arrhythmias after COVID-19 are not yet fully understood^10,12,17^. Persistent cardiac arrhythmias in patients after SARS-CoV-2 infection, ranging from relatively mild ones (such as transient sinus bradycardia) to potentially life-threatening conditions (such as ventricular tachyarrhythmias and sudden cardiac death), may result from various causes, including electrolyte disturbances, dehydration, primary myocardial damage, secondary cardiac lesions, and pre-existing cardiovascular disorders^12,18,19^.

This study aimed to assess whether the severity of the home course of COVID-19 affects the occurrence of cardiac arrhythmias and to identify the cardiac arrhythmias that occur after COVID-19 in non-hospitalized patients without a history of cardiovascular disease (CVD).

## Materials and methods

### Study group and inclusion and exclusion criteria

This retrospective study was based on the analysis of medical data of patients with COVID-19 treated on an outpatient basis with no prior history of cardiovascular disease included in the STOP-COVID registry of the Polish Long-Covid Cardiovascular (PoLoCOV-CVD) trial (ClinicalTrials.gov identifier—NCT05018052, the registration date 29.05.2020), which consists of 4142 patients who consulted a doctor due to persistent symptoms after COVID-19. Given the large size of the entire registry, this study includes only patients whose data were complete. The analysis included patients in the early post-COVID period who reported persistent symptoms within 4 weeks of SARS-CoV-2 infection and underwent echocardiography (ECHO), a 12-lead electrocardiogram (ECG), and 24-h Holter monitoring between 4 and 12 weeks after recovering from COVID-19. All individuals included in the study were informed in detail about the study and provided written consent to participate. Consent to conduct the study was obtained from the Bioethics Committee of the District Medical Chamber in Lodz (no. KB-0115/2021).

The study included patients aged ≥ 18 years, without a previous diagnosis of CVD, with a confirmed diagnosis of SARS-CoV-2 virus infection, who were treated on an outpatient basis for at least 14 days after the disappearance of acute clinical symptoms of disease and gave written consent to participate in the study. Outpatients did not receive any antiviral treatment; only symptomatic treatment and treatment for underlying conditions were provided. According to the guidelines of the Minister of Health in Poland, a “confirmed case” of COVID-19 infection was defined as any person with a SARS-CoV-2 infection confirmed using real-time reverse transcriptase polymerase chain reaction, regardless of the presence of clinical symptoms^20^. The exclusion criteria were as follow: hypertension, defined as a abnormal average daily blood pressure diagnosed by home blood pressure monitoring (HBPM) or ambulatory blood pressure monitoring (ABPM); anemia (hemoglobin serum level below 10 g/dl); thyroid dysfunction; history of cancer treated with cardiotoxic chemotherapy or chest radiotherapy; history of treatment with drugs with potential cardiotoxic effects e.g., corticosteroids^21^, remdesivir^22^, or others; reversible factors that could lead to a decrease in left ventricular ejection fraction (LVEF) and/or contractility disorders such as new ischemic changes in the ECG with positive markers of cardiac necrosis (troponin measured twice). Individuals taking any of the antiarrhythmic drugs were also excluded from the study.

**Fig. 1:**
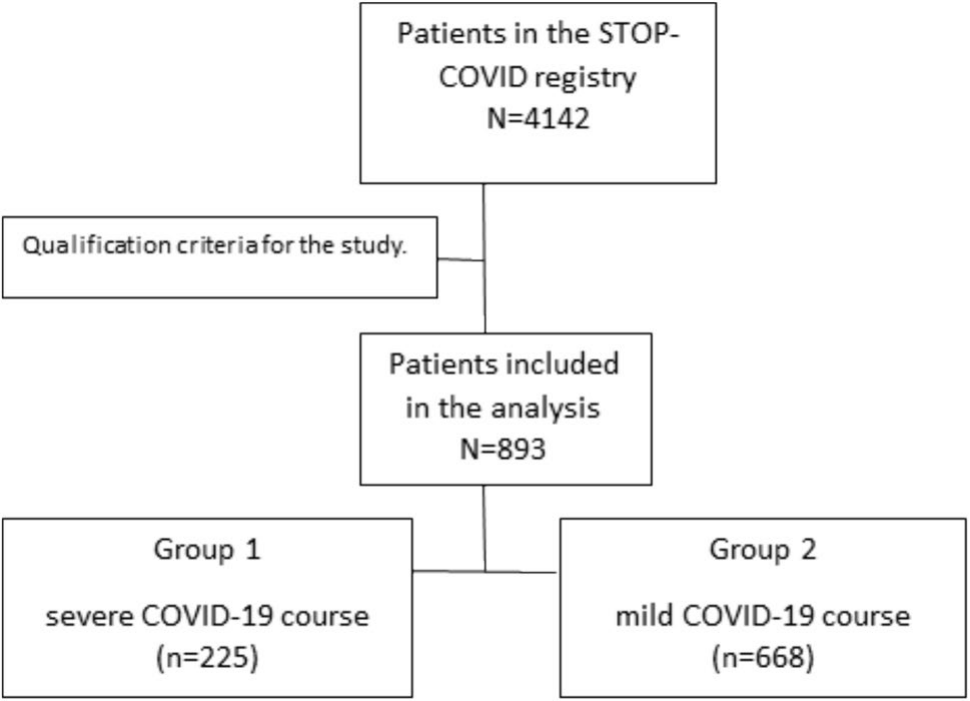
Flowchart of the study group.

Patients were divided into two groups, depending on the COVID-19 severity^23^: group 1 with a more severe course of the disease (symptoms lasting > 14 days with shortness of breath or oxygen saturation below 94% persisting for more than 3 days) and group 2 included patients with a milder course of COVID-19 (individuals not meeting the criteria to be included in group 1) – Fig. 1. All patients underwent echocardiogram (ECHO) between 4 and 12 weeks after SARS-CoV-2 infection. Myocardial damage was defined as left ventricular ejection fraction (LVEF) < 50% and/or segmental left-ventricular contractility abnormalities during echocardiography examination. Selected parameters and cardiac rhythm or conduction disorders were assessed and compared between the two study groups based on the 12-lead standard ECG and 24-h Holter ECG monitoring.

The following parameters were also assessed: gender, age, body mass index (BMI; calculated according to the formula: BMI = weight [kg] / height [m]^2^) and comorbidities (diabetes mellitus, venous thromboembolism, chronic obstructive pulmonary disease, asthma, hyperlipidemia). Each patient completed a questionnaire containing questions about clinical symptoms during the COVID-19 course. As part of the follow-up visit at 4–12 weeks after recovering from COVID-19, they completed a questionnaire regarding persistent symptoms after COVID-19. The questionnaire consisted of questions about the most common clinical symptoms, identified among the long-COVID ailments that occur after 4 weeks from the onset of SARS-CoV-2 infection^24^.

### 12-lead electrocardiogram (ECG)

The ECG was recorded with the subject in a supine position, during quiet respiration at a rate of 25 mm/s. All ECGs were evaluated by an experienced clinician with over 10 years of experience in the field of cardiology. The following parameters were collected and analyzed on the standard ECG: mean heart rate (mHR; bpm), PQ interval (PQ; msec.), QRS interval (QRS; msec.), QTc interval (msec.) with the Bazett formula in lead II^25^, ST-T segment and/or T wave (STT; msec.), and QRS fragmentation (fQRS), defined as the presence of various morphologies of the QRS wave with or without a Q wave, which includes the presence of an additional R wave (R') or notching in the nadir of the R’ (fragmentation) in two contiguous leads, corresponding to a major coronary artery territory^26^.

An abnormality was considered to be the presence in a standard resting 12-lead ECG of at least one of the following parameters: sinus tachycardia (> 100 bpm), QRS complex duration > 120 ms., second- and third-degree AV block, prolonged QTc interval > 450 ms. (patients with medications which might prolong the QT interval were excluded from the study), ST-T segment elevation or depression and/or T wave abnormalities (inversion or tall T-wave), QRS fragmentation (fQRS), atrial fibrillation or flutter (AF/AFl), any heart rhythm disturbance defined as supraventricular extrasystole (SVES) and/or ventricular extrasystole (VES).

### 24-h Holter monitoring

24-h ECG Holter monitoring was performed with PocketECG III (Medicalgorithmics Unified Arrhythmia Diagnostic System, Warsaw, Poland). The following parameters and heart rate disturbances were assessed: (1) minimal heart rate (bpm); (2) maximal heart rate (bpm); (3) mean heart rate (bpm); (4) number of premature supraventricular extrasystole (SVES); (5) number of supraventricular tachycardia other than atrial fibrillation/flutter (any episodes of tachycardia supraventricularis (TSV) lasting > 30 s); (6) number of atrial fibrillation/flutter episodes lasting > 30 s); (7) number of premature VES; (8) number of non-sustained ventricular tachycardia episodes ≥ 3 consecutive ventricular beats at ≥ 120 bpm lasting < 30 s (nsVT); and (9) number of sustained ventricular tachycardia (VT); (10) second-degree Wenckebach, second-degree Mobitz, third-degree AV block. A significant ventricular arrhythmia was defined as the occurrence of a VES > 1000 / 24 h and/or any episode of nsVT. Any heart rhythm abnormalities in 24-h ECG Holter was defined as the prevalence of at least one of the following parameters: mean heart rate > 90 bpm and/or atrial fibrillation, number of SVES > 1000, any episode of TSV, number of VES > 1000, any episode of nsVT, any episode of block A-V second degree type 2 and/or any episode block A-V third degree^27^.

### Evaluation of echocardiography

The echocardiography procedure was conducted by an experienced cardiologist, in accordance with the European Association of Cardiovascular Imaging (EACVI) recommendations^28^. To ensure a comprehensive examination, standard parasternal and apical projections were recorded (four-chamber, two-chamber, and three-chamber), as well as a modified apical projection for the right ventricle. These projections enabled the visual analysis of regional contractility in 17 segments of the left ventricular muscle, as well as the contractile function of the right ventricle.

Quantitative measurements of left ventricular function were obtained on the basis of the modified Simpson method. The assessment of myocardial dysfunction was based on left ventricular ejection fraction (LVEF) < 50% and/or segmental left-ventricular contractility abnormalities during echocardiography examination. Quantitative assessment of the right ventricular systolic function was based on the evaluation of the tricuspid annular systolic excursion amplitude (TAPSE) and the measurement of the maximum myocardial systolic velocity S’ of the tricuspid annulus/basal segment of the RV free wall, determined using tissue echocardiography (TDE). Transvalvular flows were determined using colour, pulsed, and continuous Doppler imaging. The mitral inflow profile was evaluated, and estimates of pulmonary pressures were obtained.

### Statistical analysis

The analyzed variables were both qualitative and quantitative. The normality of distribution was assessed using the Shapiro–Wilk test. Differences between qualitative variables were assessed using the chi-square test. For quantitative variables, the non-parametric Mann–Whitney U-test was used. To identify risk factors for the development of significant ventricular arrhythmia, univariate logistic regression models were constructed, where the dependent variable was the occurrence of significant ventricular arrhythmia, and the independent variables included sociodemographic data, the course of COVID-19 severity, Holter ECG features, as well as symptoms during COVID-19 and long-COVID. In the next stage, a complex backward stepwise logistic regression model was constructed, with analogous calculations performed for the dependent variable (ventricular arrhythmia) and the independent variables, which included specific parameters from echocardiography, sociodemographic data, and symptoms experienced during COVID-19 and long-COVID. The analysis was carried out using Statistica 13.0 by StatSoft. A *p*-value < 0.05 was assumed to be statistically significant.

## Results

### Characteristics of the study group

The analysis included a total of 893 patients (66% women and 34% men), with an average age of 46.5 ± 12.4 years and encompassing 225 patients (25.2%) with severe COVID-19. Both in Group 1 and Group 2, women were significantly more frequent (*p* = 0.043). Moreover, a statistically significant difference was observed between the study groups in terms of BMI (*p* = 0.024). No statistically significant difference was observed in the occurrence of comorbidities (*p* = 0.129); however, among chronic diseases, asthma occurred significantly more often in the group with severe COVID-19 (*p* = 0.039). Table 1 presents a detailed summary of the included non-hospitalized COVID-19 patients divided into the study groups. A detailed summary of the COVID-19 symptoms and severity, as well as long-COVID symptoms, is presented in the supplementary material (Table S1).

**Table 1:** The clinical characteristics of the study groups.

Parameters / variables		Group 1 severe COVID-19 course	Group 2 mild COVID-19 course	*p*-value	All patients included
No. of patients		225	668		893
Sex	Male	60 (26.67%)	248 (37.13%)	0.043#	308 (34.49%)
	Female	165 (73.33%)	420 (62.87%)		585 (65.51%)
Age		46.69 ± 11.23	46.47 ± 12.71	0.654*	46.53 ± 12.35
Weight [kg]		76.69 ± 17.29	75.61 ± 15.98	0.653*	75.88 ± 16.32
Height [cm]		168.72 ± 7.78	170.69 ± 8.75	0.007*	170.20 ± 8.56
BMI [kg/m^2^]		26.84 ± 5.44	25.86 ± 4.65	0.024*	26.11 ± 4.88
DM		7 (3.11%)	17 (2.54%)	0.649#	24 (2.69%)
Hyperlipidemia		28 (12.44%)	74 (11.08%)	0.576#	102 (11.42%)
Asthma		26 (11.56%)	48 (7.19%)	0.039#	74 (8.29%)
COPD		2 (0.89%)	3 (0.25%)	0.444#	5 (0.56%)
Presence of at least one chronic disease		146 (64.89%)	470 (70.36%)	0.129#	616 (68.98%)

*BMI* Body Mass Index, *DM* diabetes mellitus, *COPD* chronic obstructive pulmonary disease, * Mann–Whitney U-Test; # Chi-squared test.

### 12-lead ECG and 24-h holter ECG

The analysis of parameters from the 12-lead ECG revealed a statistically significant difference between the study groups in terms of the duration of the QRS complex (*p* = 0.035), while the analysis of the 24-h ECG monitoring parameters showed the more frequent occurrence of couples of supraventricular extrasystoles in Group 1 (*p* = 0.037). Despite statistically significant differences in the QRS duration and SVES parameters, their clinical significance is doubtful as the values fall within the normal range. Both in the ECG recording and in 24-h ECG monitoring, atrial fibrillation occurred in a very small percentage of patients. There were no differences between groups in the percentage of ventricular life-threatening arrhythmias and/or atrioventricular conduction disorders. The results regarding ECG abnormalities and cardiac arrhythmias are summarized in Tables 2 and 3. No statistically significant differences were observed between the two studied groups in any of the analyzed echocardiographic parameters. A detailed summary of the tested parameters is presented in the supplementary material (Table S2).

**Table 2:** 12-lead ECG results among non-hospitalized patients with mild or severe course of COVID-19.

Parameters / variables	Group 1—severe COVID-19 course (n = 225)	Group 2—mild COVID-19 course (n = 668)	*p*-value	All included patients (n = 893)
12-lead ECG parameters—average values and standard deviation				
Mean heart rate (mHR) [bpm]	75.33 ± 12.77	75.43 ± 12.26	0.754*	75.40 ± 12.38
PR interval [msec.]	158.32 ± 21.90	157.28 ± 25.43	0.264*	157.54 ± 24.58
QRS duration [msec.]	92.20 ± 12.88	94.15 ± 12.75	0.035*	93.66 ± 12.80
QTcB [msec.]	413.52 ± 25.33	411.64 ± 24.99	0.471*	412.11 ± 25.08
12-lead ECG parameters—number and percentage of patients				
Sinus rhythm	224 (99.56%)	667 (99.85%)	0.419#	891 (99.78%)
Atrial fibrillation	1 (0.44%)	1 (0.15%)	0.419#	2 (0.22%)
PQ > 200 [msec.]	6 (2.67%)	22 (3.29%)	0.641#	28 (3.14%)
QRS > 100 [msec.]	42 (18.67%)	160 (23.95%)	0.101#	202 (22.62%)
QRS ≥ 120 [msec.]	9 (4.00%)	26 (3.89%)	0.943#	35 (3.92%)
QTc > 460F > 450 M [msec.]	14 (6.22%)	23 (3.44%)	0.070#	37 (4.14%)
STT abnormal	18 (8.00%)	53 (7.93%)	0.975#	71 (7.95%)
T wave inverted	11 (4.89%)	33 (4.94%)	0.976#	44 (4.93%)
ST-T abnormalities or T wave inverted	18 (8.00%)	54 (8.08%)	0.968#	72 (8.06%)
LBBB	1 (0.44%)	3 (0.45%)	0.993#	4 (0.45%)
RBBB	9 (4.00%)	26 (3.89%)	0.943#	35 (3.92%)
IST (mHR) > 100 bpm	7 (3.11%)	23 (3.44%)	0.812#	30 (3.36%)
SVES	1 (0.44%)	10 (1.50%)	0.215#	11 (1.23%)
VES	6 (2.67%)	14 (2.10%)	0.616#	20 (2.24%)
Arrhythmias (AF, AFl, SVES, VES)	3 (1.33%)	20 (2.99%)	0.174#	23 (2.58%)
QRS fragmentation	40 (17.78%)	115 (17.22%)	0.847#	155 (17.36%)
Any abnormality (HR > 100, QRS ≥ 120 ms, ST-T changes, T changes, arrhythmia)	72 (32.00%)	215 (32.19%)	0.959#	287 (32.14%)

*mHR* mean heart rate; *PR* PR interval, *QRS* QRS complex, *QTcB* QTc interval with the Bazett formula in lead II, *PQ* PQ interval, *ST* ST segment, *AF* atrial fibrillation, *LBBB* left bundle branch block, *RBBB* right bundle branch block, *IST* inappropriate sinus tachycardia, *SVES* supraventricular extrasystole, *VES* ventricular extrasystole, *ECG* electrocardiogram, *Stach sinus tachycardia, *Min* minimal, *Max* maximal, *F* female, *M* male Mann–Whitney U-Test, # Chi-squared test.

**Table 3:** 24-h Holter ECG results among non-hospitalized patients with mild or severe course of COVID-19.

24-h Holter ECG—average values and standard deviation				
HR min. [bpm]	57.52 ± 8.47	57.23 ± 8.21	0.724*	57.30 ± 8.27
HR max. [bpm]	130.52 ± 17.38	132.51 ± 19.25	0.137*	132.01 ± 18.80
HR mean [bpm]	74.91 ± 9.92	75.51 ± 9.12	0.464*	75.36 ± 9.33
No. of SVES / 24 h	20.87 ± 104.91	83.50 ± 840.69	0.448*	67.72 ± 729.37
No. of pairs SVES / 24 h	0.10 ± 0.39	0.37 ± 2.16	0.037*	0.30 ± 1.88
No. of TSV / 24 h	0.16 ± 0.81	0.83 ± 14.33	0.103*	0.66 ± 12.40
No. of VES / 24 h	168.36 ± 1111.69	123.95 ± 946.04	0.450*	135.14 ± 989.93
No. of pairs of VES / 24 h	0.49 ± 5.08	0.24 ± 2.07	0.631*	0.31 ± 3.12
No. of nsVT / 24 h	0.04 ± 0.47	0.01 ± 0.09	0.833*	0.02 ± 0.25

24-h Holter ECG—number and percentage of patients				
STach (mHR > 90 bpm)	13 (5.78%)	29 (4.34%)	0.378#	42 (4.70%)
SVES > 1000	1 (0.44%)	10 (1.50%)	0.215#	11 (1.23%)
SVT	14 (6.22%)	66 (9.88%)	0.096#	80 (8.96%)
AF	0 (0.0%)	1 (0.15%)	0.561#	1 (0.11%)
VES > 1000 / 24 h	6 (2.67%)	14 (2.10%)	0.616#	20 (2.24%)
nsVT / 24 h	2 (0.89%)	5 (0.75%)	0.836#	7 (0.78%)
VES > 1000 and/or nsVT	7 (3.11%)	19 (2.84%)	0.837#	26 (2.91%)
AV block 2nd degree type 2 and/or block 3rd degree	1 (0.44%)	2 (0.30%)	0.746#	3 (0.34%)
Any heart rhythm abnormality	37 (16.44%)	124 (18.56%)	0.474#	161 (18.03%)

*mHR* mean heart rate, *AF* atrial fibrillation, *SVES* supraventricular extrasystole, *VES* ventricular extrasystole, *ECG* electrocardiogram, *SVT* supraventricular tachycardia, *nsVT* non-sustained ventricular tachycardia, *AV* atrioventricular, *Stach sinus tachycardia, *Min* minimal, *Max* maximal, *F* female, *M* male Mann–Whitney U-Test, # Chi-squared test.

Univariate regression analysis revealed that age (*p* < 0.001), ejection fraction (EF) (*p* = 0.01), and cardiac dysfunction (EF < 50% and/or any contraction abnormalities) (*p* < 0.001) were associated with a higher risk of myocardial damage in the post-COVID-19 period. A detailed summary of the univariate analysis results is presented in Table S3. Multivariate regression analysis demonstrated that age (*p* = 0.018) and cardiac dysfunction (*p* = 0.008) were associated with an increased risk of cardiac arrhythmias in patients with COVID-19 (Table 4).

**Table 4:** Backward stepwise logistic regression model for significant ventricular arrhythmia.

Parameters / variables	Significant ventricular arrhythmia (VES > 1000 / 24 h and/or any episode of nsVT)	
	OR [95%CI]	*p*-value
Age	1.04 [1.01–1.08]	0.018
Cardiac dysfunction (EF < 50% and/or contractile dysfunction)	3.52 [1.39–8.96]	0.008

*OR* odds ratio, *CI* confidence interval, *VES* ventricular extrasystole, *nsVT* non-sustained ventricular tachycardia, *EF* ejection fraction, *ECHO* echocardiography.

### 24-h Holter ECG monitoring and evaluation of echocardiography

The 24-h Holter ECG-related univariable analysis of ECHO parameters, long-COVID symptoms, COVID-19 symptoms and severity, or socioeconomic parameters showed that systolic LV (*p* = 0.005), heart palpitations (*p* = 0.016), hyperlipidemia (*p* = 0.004), age (*p* < 0.001), and the presence of at least one chronic disease (*p* < 0.001) were associated with a higher risk of cardiac arrhythmias after COVID-19, respectively. A detailed summary of the univariate analysis results is presented in Table S4. Multivariate regression analysis showed that age (*p* < 0.001), systolic LV (*p* < 0.001), diastolic LV (*p* < 0.001), LA diameter (*p* = 0.006), and the presence of at least one chronic disease (*p* = 0.014) were significantly associated with the occurrence of cardiac arrhythmias in non-hospitalized patients after COVID-19 (Table 5).

**Table 5:** Multivariate analysis related to 24-h Holter ECG.

Parameters / variables	Significant ventricular arrhythmia (VES > 1000 / 24 h and/or any episode of nsVT)	
	OR [95%CI]	*p*-value
Age	1.05 [1.02–1.05]	< 0.001
LV systolic diameter	0.93 [0.85–0.95]	< 0.001
LV diastolic diameter	1.10 [1.06–1.21]	< 0.001
LA diameter	0.94 [0.88–0.98]	0.006
Presence of at least one chronic disease	2.21 [1.11–2.43]	0.014

*OR* odds ratio, *CI* confidence interval, *VES* ventricular extrasystole, *nsVT* non-sustained ventricular tachycardia, *LV* left ventricle, *LA* left atrium.

## Discussion

The research shed light on the prevalence of cardiac arrhythmias in non-hospitalized COVID-19 patients without a history of cardiovascular diseases and their association with the severity of the disease. The study included 893 non-hospitalized patients who had recovered from COVID-19, divided into two groups based on disease severity (Group 1 and Group 2). The patients underwent electrocardiogram (ECG) and 24-h Holter ECG tests in the post-COVID period. Various cardiac parameters, including heart rate, intervals, arrhythmias, and syndromes, were analyzed and compared between the groups.

In our study, women were significantly more frequent in both Group 1 and Group 2. Recent studies conducted on a large group of patients confirmed that female gender increases the risk of developing post-COVID-19 syndrome^29,30^. Furthermore, other multicenter studies have also shown that female gender increases the likelihood of developing cardiopulmonary post-COVID-19 symptoms, such as fatigue or shortness of breath^31,32^. In the Loosen et al. study^33^, based on data from 50,402 COVID-19 patients in the Disease Analyzer database (IQVIA, Germany), lipid metabolism disorders and obesity were identified as strong risk factors for developing long COVID-19. Additionally, in the Metabolic Syndrome in Active Subjects (MESYAS) study of 18,778 patients, a ratio of triglycerides to high-density lipoprotein greater than 2.75 in men and greater than 1.65 in women was found to be highly predictive of metabolic syndrome diagnosis. The TG/HDL ratio was also found to have a high predictive value for a first coronary event, regardless of body mass index^34^.

Our study revealed that BMI is higher in the group of patients with a more severe course of disease (Group 1). Moreover, the same individuals were more likely to have asthma. A systematic review by Nagar et al. indicated that morbid obesity is linked to mortality of COVID-19 patients and that increased BMI is positively related to the disease^35^. As for asthma, whether it increases the risk of severe COVID-19 is unclear; discrepancies in the literature are caused by many aspects, such as the lack of uniform asthmatic populations, the obedience of the asthmatic population, distinct rates of infection, the local PCR testing policy, and disparate vulnerability to SARS-CoV-2 infection in various ethnic groups. Sunjaya et al. concluded that asthma is not an independent factor for severe COVID-19^36^. Dolby et al. concluded that individuals with poorly controlled or severe asthma were at increased risk of hospitalization due to COVID-19 compared to individuals without asthma. However, this association did not persist in the study of mild or well-controlled asthma. Asthma is associated with cardiovascular disease, and individuals affected by these conditions often have an increased risk of mortality^37^.

Multivariate regression analysis identified age, systolic LV diameter, diastolic LV diameter, LA diameter, and the presence of at least one chronic disease as independent factors significantly associated with the occurrence of cardiac arrhythmias. Indeed, the risk of cardiac arrhythmias after COVID-19 was particularly increased among older individuals and patients suffering from a severe course of disease^38,39^. Myocardial contractile dysfunction is known to be a manifestation of cardiac complications related to COVID-19^40^. Other research indicated that abnormal left ventricular global systolic longitudinal strain (LV-GLS), which thoroughly assesses LV systolic function^41^, is significantly related to the incidence of arrhythmias requiring intervention^42^. Moreover, diastolic dysfunction was observed twice as often in the group of patients three months after hospitalization for COVID‐19 than in matched controls; cardiac arrhythmias were common after hospitalization, with premature ventricular beats present in every fifth patient^43^. Zylla et al. hinted at a putative role of reduced LVEF as a risk factor for arrhythmia during hospitalization due to COVID-19^44^. Ergül and colleagues concluded that functions of the left atrium may be impaired among COVID-19 convalescents^45^, which corresponds to the Goerlich et al. study indicating diminished LA function in hospitalized COVID-19 patients relative to COVID-19-negative controls with similar degrees of critical illness, as well as a more pronounced dysfunction among COVID-19 patients who develop atrial fibrillation^46^. Lastly, it was previously confirmed that COVID-19 patients with cardiac arrhythmia have a higher frequency of comorbidities, including cardiovascular ones^14,47^.

It is worth noting other clues from our study. For example, there is a scarcity of individuals with atrial fibrillation relative to our previous publication on hospitalized COVID-19 patients^48^. Numerous studies have shown that atrial fibrillation is common following COVID-19^49,50^; however, these studies concern hospitalized patients and the acute period after COVID-19. In our study, the follow-up period was extended (at least 4 weeks after SARS-CoV-2 infection), and patients had no history of cardiovascular disease or significant LV dysfunction, which may explain the rare occurrence of AF episodes. Furthermore, recognition of significant ventricular arrhythmia (VES > 1000/d and/or any episode of nsVT) should be an indication to expand the diagnostics and perform ECHO, since the high risk of left ventricular dysfunction occurs even among patients after a mild home course of COVID-19 and without significant cardiovascular disease. Due to the fact that the presence of certain chronic diseases increases the risk of arrhythmias, affected people are at risk of complications after COVID-19 and thus require greater clinical vigilance. Therefore, one of the tests that should be considered in patients with symptoms after COVID-19 is an ECG (a simple and easily accessible test, even in primary healthcare), which can indicate heart muscle damage-related abnormalities that require further diagnostic evaluation. The literature also includes information on the Index of cardiac electrophysiological balance (iCEB), which is a relatively new electrocardiographic parameter that reflects the balance between ventricular depolarization and repolarization. It can also be used to monitor arrhythmias in patients undergoing short-term treatment for COVID-19^51^. In general, further studies are needed to examine a broader patient group, taking into account demographic differences and ethnicity.

We acknowledge that our study had limitations. It did not include all patients affected by COVID-19 because it focused on patients who self-presented to a medical facility – this potentially overrepresented symptomatic individuals and underestimates the true prevalence of arrhythmias in the general post-COVID population. The absence of a non-COVID control group limits the ability to determine whether the observed arrhythmia rates are elevated compared to the general population. The study also did not assess vaccination status related to influenza and COVID-19. In our study, disease severity was evaluated retrospectively using medical records and questionnaire data. The definition of “severe” COVID-19 (symptoms persisting for more than 14 days with dyspnea or SpO₂ < 94% for more than 3 days) may not align with standard severity classifications and appears relatively mild compared to the WHO criteria. Moreover, many people may not have been hospitalized even though they were eligible; the reasons may have included a refusal due to fear of being hospitalized or a lack of available hospital beds. This could have potentially led to the inclusion of patients who actually required hospitalization in the “non-hospitalized” group. The wide range of follow-up timing (4–12 weeks) could introduce variability in outcomes, as cardiac effects may evolve differently over time. Unfortunately, we do not have data on viral load, inflammatory marker levels, or markers of myocardial damage (such as troponin). The study only examines the early post-COVID period; longer-term cardiac consequences remain unknown. Furthermore, when interpreting multivariate regression results, caution is required regarding the independent contributions of the selected parameters, as the model includes the “presence of at least one chronic disease,” which is an extensive category. Nevertheless, the study’s strength lies in the compilation of data obtained from the STOP-COVID registry, which is one of the largest Polish databases concerning COVID-19 patients, most of whom were treated on an outpatient basis due to SARS-CoV-2 infection. Focusing on non-hospitalized patients without prior CVD addresses a critical gap, as most previous studies examined hospitalized patients. The exclusion of patients with hypertension, anemia, thyroid dysfunction, and cardiotoxic medications helps isolate COVID-19-related effects. This study enables the assessment of the impact of COVID-19 on cardiac arrhythmias among patients without hospitalization. It also enables the thorough identification of predictors of myocardial damage in the ECG and/or 24-h Holter monitoring, facilitating early diagnosis by a primary care physician and patient referral for further cardiological diagnostics. It is crucial because many patients infected with SARS-CoV-2 and with a mild course of the disease are treated at home.

## Conclusions

The occurrence of cardiac arrhythmias among non-hospitalized patients without a prior diagnosis of cardiovascular disease does not depend on the severity of COVID-19. Identifying significant ventricular arrhythmia (VES > 1000 / 24 h and/or any episode of nsVT) should be a sign to extend the diagnostics and perform ECHO because the risk of left ventricular dysfunction is high even among patients after a mild home course of COVID-19 and without significant cardiovascular diseases. One of the tests that should be considered in patients with symptoms after COVID-19 is an ECG, which can indicate heart muscle damage-related abnormalities that require further diagnostics.

## Supplementary Information

Supplementary Information.

## Data Availability

The data presented in this study are available on request from the corresponding author.

## Abbreviations

PoLoCOV-CVD: Polish long-COVID cardiovascular
SARS-CoV-2: Severe acute respiratory syndrome coronavirus 2
COVID-19: Coronavirus disease 2019
CVD: Cardiovascular disease
ECG: Electrocardiogram
HBPM: Home blood pressure monitoring
ABPM: Ambulatory blood pressure monitoring
LVEF: Left ventricular ejection fraction
ECHO: Echocardiogram
BMI: Body mass index
mHR: Mean heart rate
fQRS: QRS fragmentation
AF: Atrial fibrillation
AFl: Atrial flutter
SVES: Supraventricular extrasystole
VES: Ventricular extrasystole
TSV: Tachycardia supraventricularis
nsVT: Non-sustained ventricular tachycardia
EACVI: European association of cardiovascular imaging
TAPSE: Tricuspid annular systolic excursion amplitude
TDE: Tissue echocardiography
DM: Diabetes mellitus
COPD: Chronic obstructive pulmonary disease
PR: PR interval
QRS: QRS complex
QTcB: QTc interval with the Bazett formula in lead II
PQ: PQ interval
ST: ST segment
LBBB: Left bundle branch block
RBBB: Right bundle branch block
IST: Inappropriate sinus tachycardia
SVT: Supraventricular tachycardia
AV: Atrioventricular
Stach: Sinus tachycardia
Min: Mininal
Max: Maximal
F: Female
M: Male
OR: Odds ratio
CI: Confidence interval
EF: Ejection fraction
ECHO: Echocardiography
LV: Left ventricle
LA: Left atrium
LV-GLS: Left ventricular global systolic longitudinal strain
RR: Respiratory rate
HA: Arterial hypertension
IV: Interventricular
RV: Right ventricle
LVM: Left ventricle mass
VTE: Venous thromboembolic events
BP: Blood pressure
EHRA: European heart rhythm association

## Declarations

### Clinical trial

Our retrospective study was based on the analysis of medical data from patients with COVID-19 treated on an outpatient basis in the STOP-COVID registry of the Polish Long-COVID Cardiovascular (PoLoCOV-CVD) study (ClinicalTrials.gov identifier: NCT05018052, registration date: 29 May 2020).
