# Supplementary material for: Risk assessment of cardiac arrhythmias in the early post-COVID-19 period in non-hospitalized patients—long-term data from the PoLoCOV-CVD study
Source: Sci Rep. 2026 Apr 16;16:17661. doi: 10.1038/s41598-026-47954-0 (PMC13243599; doi:10.1038/s41598-026-47954-0)
Supplement: Supplementary file 1 — Supplementary Information. [file 41598_2026_47954_MOESM1_ESM.docx]

**Supplementary materials**

**Table S1. COVID-19 symptoms and severity, as well as long-COVID symptoms.**

| **Parameters / variables** | | **All included patients (n=893)** |
| --- | --- | --- |
| **Symptoms and severity of COVID-19** | | |
| Temperature < 36.6°C | | 116 (12.99%) |
| Temperature < 37.5°C | | 218 (24.41%) |
| Temperature > 37.5°C | | 444 (49.72%) |
| Cough | | 541 (60.58%) |
| Dyspnoea | | 380 (42.55%) |
| Weakness | | 643 (72.01%) |
| Chest pain | | 416 (46.59%) |
| Diarrhea | | 167 (18.70%) |
| Vomiting | | 59 (6.61%) |
| Chills | | 322 (36.06%) |
| Hearing loss/decreased hearing | | 87 (9.74%) |
| Smell and/or taste disorders | | 430 (48.15%) |
| Arthralgia | | 346 (38.75%) |
| High RR and/or dysregulation of well-controlled HA | | 80 (8.96%) |
| Course of disease | 0 – mild at home | 668 (74.80%) |
|  | 1 – severe at home | 225 (25.20%) |
| **Symptoms of long-COVID** | | |
| Weakness | | 566 (63.38%) |
| Worse exercise tolerance | | 530 (59.35%) |
| Cough | | 209 (23.40%) |
| Dyspnea | | 231 (25.87%) |
| Taste disturbances | | 156 (17.47%) |
| Excessive sweating | | 245 (27.44%) |
| Chest pain | | 336 (37.63%) |
| Muscle pains | | 181 (20.27%) |
| Swelling | | 76 (8.51%) |
| Skin lesions | | 56 (6.27%) |
| Memory/concentration disorders | | 393 (44.01%) |
| Headache | | 285 (31.92%) |
| Arthralgia | | 8 (0.90%) |
| Abbreviations: RR – respiratory rate; HA – arterial hypertension | | |

**Table S2. Echocardiogram results in non-hospitalized patients with mild or severe course of COVID-19.**

| **Variable parameters in echocardiography (ECHO)** | **Group 1 – severe COVID-19 course (n=225)** | | **Group 2 – mild COVID-19 course (n=668)** | | **p-value** | **All included patients (n=893)** |
| --- | --- | --- | --- | --- | --- | --- |
| **ECHO parameters** | | | | | | |
| LV systole diameter [mm] | 30.33±5.12 | | 30.15±4.91 | 0.973* | | 30.20±4.96 |
| LV diastole diameter [mm] | 44.70±4.22 | | 45.11±4.53 | 0.476* | | 45.01±4.45 |
| LA diameter [mm] | 37.52±4.38 | | 37.26±4.62 | 0.344* | | 37.32±4.56 |
| Aorta [mm] | 29.91±3.88 | | 30.20±3.79 | 0.638* | | 30.12±3.81 |
| IV Systole [mm] | 12.99±1.17 | | 13.00±1.33 | 0.833* | | 13.00±1.29 |
| IV Diastole [mm] | 9.68±1.36 | | 9.71±1.51 | 0.895* | | 9.70±1.47 |
| RV [mm] | 27.91±2.85 | | 27.93±3.81 | 0.637* | | 27.92±3.59 |
| LVM [mm] | 167.66±48.90 | | 171.14±53.83 | 0.740* | | 170.26±52.63 |
| EF [%] | 59.42±2.36 | | 59.53±2.91 | 0.344* | | 59.50±2.78 |
| TAPSE [mm] | 25.51±2.35 | | 25.44±2.41 | 0.704* | | 25.45 ± 2.39 |
| Cardiac dysfunction  (EF<50% and/or contractile dysfunction on ECHO) | no | 203 (90.22%) | 610 (91.32%) | 0.619# | | 813 (91.05%) |
|  | yes | 22 (9.78%) | 58 (8.68%) |  |  | 80 (8.95%) |
| Abbreviations: ECG – electrocardiogram; LV – left ventricle; LA – left atrium; IV – interventricular; RV- right ventricle; LVM -left ventricle mass; EF - ejection fraction; TAPSE - Tricuspid Annular Plane Systolic Excursion; ECHO – echocardiography, * Mann-Whitney U Test, # Chi-squared test | | | | | | |

**Table S3. Significant ventricular arrhythmia – univariate analysis.**

| **Variable** | | **Significant ventricular arrhythmia** | |
| --- | --- | --- | --- |
|  |  | **OR [95%CI]** | **p-value** |
| **COVID-19 severity and socioeconomic parameters** | | | |
| COVID-19 course | Group 1 – severe home course | 1.10 [0.46, 2.64] | 0.838 |
| Gender | Female | 0.61 [0.28, 1.32] | 0.209 |
| Age | | 1.05 [1.02, 1.08] | **<0.001** |
| Comorbidities | Diabetes mellitus | 3.20 [0.71, 14.39] | 0.128 |
|  | Hyperlipidemia | 1.01 [0.30, 3.43] | 0.993 |
|  | Asthma | 0.44 [0.06, 3.26] | 0.421 |
|  | COPD | 8.63 [0.93, 80.03] | 0.062 |
| Presence of at least one chronic disease | | 1.66 [0.75, 3.66] | 0.207 |
| **Echocardiographic parameters** | | | |
| LV systolic [mm] | | 1.01 [0.93, 1.10] | 0.758 |
| LV diastolic [mm] | | 1.05 [0.96, 1.14] | 0.309 |
| LA [mm] | | 1.00 [0.91, 1.09] | 0.938 |
| EF [%] | | 0.89 [0.82, 0.97] | **0.008** |
| Cardiac dysfunction (EF<50% and/or  contractile dysfunction on ECHO) | | 4.01 [1.63, 9.85] | **<0.001** |
| **COVID-19 symptoms** | | | |
| Dyspnoea | | 1.36 [0.62, 2.97] | 0.438 |
| Weakness | | 1.65 [0.62, 4.44] | 0.318 |
| Chest pain | | 1.59 [0.72, 3.49] | 0.248 |
| High RR and/or dysregulation  of well-controlled HA | | 0.40 [0.05, 2.98] | 0.368 |
| **Long-COVID symptoms** | | | |
| Heart palpitations: moderate, severe, or disabling (according to EHRA symptoms scale ^38^) | | 1.43 [0.65, 3.14] | 0.381 |
| Fainting/loss of consciousness | | 0.90 [0.12, 6.80] | 0.923 |
| Worse exercise tolerance | | 1.89 [0.79, 4.55] | 0.163 |
| Dyspnoea | | 1.83 [0.82, 4.09] | 0.142 |
| Chest pain | | 0.87 [0.39, 1.98] | 0.754 |
| Abbreviations: OR – odds ratio; VTE – venous thromboembolic events; COPD – chronic obstructive pulmonary disease; LV – left ventricle; LA – left atrium; EF – ejection fraction; ECHO – echocardiography; BP – blood pressure; HA – arterial hypertension; EHRA – European Heart Rhythm Association | | | |

**Table S4. Univariate analysis related to 24-hour Holter ECG.**

| **Parameters / variables** | **Any abnormality in the 24-hour Holter ECG** | |
| --- | --- | --- |
|  | **OR [95% CI]** | **p-value** |
| **24-hour Holter ECG and ECHO** | | |
| Cardiac dysfunction (EF<50% and/or contractile dysfunction on ECHO) | 1.15 [0.65, 2.05] | 0.631 |
| LV systolic | 0.95 [0.91, 0.98] | **0.005** |
| LV diastolic | 1.00 [0.96, 1.04] | 0.983 |
| LA | 0.96 [0.93, 1.00] | 0.066 |
| LV EF | 0.93 [0.93, 1.05] | 0.671 |
| **24-hour Holter ECG and long-COVID** | | |
| Heart palpitations: moderate, severe, or disabling (according to EHRA symptoms scale ^38^) | 1.53 [1.08, 2.15] | **0.016** |
| Fainting/loss of consciousness | 0.68 [0.26, 1.77] | 0.427 |
| Worse exercise tolerance | 1.23 [0.86, 1.75] | 0.254 |
| Dyspnoea | 1.05 [0.72, 1.55] | 0.788 |
| Chest pain | 0.78 [0.54, 1.12] | 0.174 |
| **24-hour Holter ECG and COVID-19 symptoms or disease severity** | | |
| Temperature >37.5°C | 0.88 [0.63, 1.25] | 0.481 |
| Dyspnoea | 1.34 [0.95, 1.88] | 0.095 |
| Weakness | 1.22 [0.82, 1.80] | 0.326 |
| Chest pain | 1.16 [0.83, 1.64] | 0.383 |
| High RR and/or dysregulation of well-controlled HA | 0.87 [0.47, 1.62] | 0.665 |
| The course of the disease (severe at home) | 0.86 [0.58, 1.29] | 0.475 |
| **24-hour Holter ECG and socioeconomic parameters** | | |
| Gender (female) | 1.21 [0.84, 1.74] | 0.312 |
| VTE | 1.52 [0.30, 7.61] | 0.609 |
| Hyperlipidemia | 1.98 [1.24, 3.17] | **0.004** |
| Asthma | 1.65 [0.95, 2.86] | 0.076 |
| COPD | 1.03 [0.57, 1.56] | 0.997 |
| Age | 1.03 [1.02, 1.05] | **<0.001** |
| BMI | 1.00 [0.97, 1.04] | 0.953 |
| DM | 1.91 [0.78, 4.67] | 0.157 |
| Presence of at least one chronic disease | 1.95 [1.37, 2.77] | **<0.001** |
| Abbreviations: OR – odds ratio; CI – confidence interval; ECG – electrocardiogram; ECHO – echocardiography; LV – left ventricle; LA – left atrium; LV EF – left ventricular ejection fraction; EHRA – European Heart Rhythm Association; RR – respiratory rate; HA – arterial hypertension; VTE – venous thromboembolic events; COPD – chronic obstructive pulmonary disease; BMI – Body Mass Index; DM – diabetes mellitus. | | |
